# Mallory–Weiss syndrome complicated by severe aspiration pneumonitis in an infant

**DOI:** 10.1093/omcr/omab094

**Published:** 2021-10-26

**Authors:** Yukako Ebara, Akihiko Shimizu, Shigeru Nomura, Akira Nishi, Yoshiyuki Yamada

**Affiliations:** 1 Department of Allergy, Infectious Diseases and Immunology, Gunma Children’s Medical Center, Shibukawa, Gunma 377-8577, Japan; 2 Department of Surgery, Gunma Children’s Medical Center, Shibukawa, Gunma 377-8577, Japan

## Abstract

A 1-month-old girl presented with hematemesis and dyspnea. A large amount of blood was aspirated through a nasogastric tube, and chest computed tomography showed bilateral centrilobular opacified lesions, which suggested aspiration pneumonitis due to upper gastrointestinal bleeding. Her respiratory condition exacerbated, and we initiated nitric oxide (NO) therapy. Bleeding stopped with conservative treatment. She was weaned off mechanical ventilation and extubated on Day 6 after admission. Afterward, upper gastrointestinal endoscopy showed a longitudinal linear scar indicative of Mallory–Weiss syndrome (MWS). MWS is rarely reported in early infancy since many of the risk factors are absent in infants. Patients with aspiration pneumonitis usually recover respiratory function within 24 h and severe respiratory failure is rare in aspiration pneumonitis. There are no pediatric case reports describing MWS with severe aspiration pneumonitis. Although MWS is a rare cause of neonatal hematemesis, patients can become severely ill and require multidisciplinary treatment.

## INTRODUCTION

Mallory–Weiss syndrome (MWS) is a condition, in which a sudden increase in intragastric pressure causes laceration of the mucosa near the esophagogastric junction. It is a rare cause of hematemesis in infancy [1]. Although it can sometimes cause anemia or even shock because of severe bleeding, no case of infant with MWS complicated with severe aspiration pneumonitis has been reported. Herein, we present a case of MWS complicated by severe aspiration pneumonitis in an infant.

## CASE REPORT

**Figure 1:**
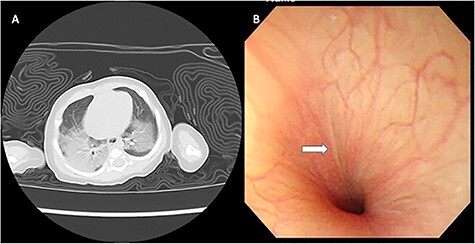
Bilateral centrilobular opacified lesions detected by chest computed tomography (A). Upper gastrointestinal endoscopy shows a longitudinal linear scar at the gastroesophageal junction (B).

A 1-month-old girl presented with hematemesis and dyspnea. Two hours after breastfeeding, she suddenly vomited a large amount of fresh blood without any identifiable cause and was transferred to a hospital. A large amount of coagulated blood was aspirated through a nasogastric tube, and chest computed tomography findings showed bilateral centrilobular opacified lesions (Fig. 1A). The patient was diagnosed with aspiration pneumonitis due to upper gastrointestinal bleeding.

As acute respiratory failure progressed, she was intubated to secure the airway, and mechanical ventilation was commenced. Her hemoglobin level was 8.7 g/dL, and venous blood gas analysis showed mixed acidosis (pH, 7.277; partial pressure of carbon dioxide, 49.5 torr; bicarbonate level, 22.6 mmol/L; base excess, −4.3 mEq/L). Red blood cells were transfused, and she was transferred to our hospital for further medical care.

The patient was born via normal vaginal delivery at 40 weeks of gestation. No abnormalities were noted during the perinatal period. She seldom vomited after birth. On admission, her body temperature, heart rate, blood pressure, and percutaneous oxygen saturation on mechanical ventilation were 36.7°C, 170 bpm, 75/42 mm Hg, and 96%, respectively. Her breathing sound decreased bilaterally. Her white blood cell count, serum C-reactive protein level, and platelet count were 13 200/μl, 0.72 mg/dl, and 293 000/μl, respectively. Coagulation tests were unremarkable.

Her respiratory condition rapidly exacerbated and hypercapnia progressed, which were suggestive of acute respiratory distress syndrome because of aspiration pneumonitis. We initiated nitric oxide (NO) therapy to treat pulmonary hypertension, because ultrasonography findings showed features of increased right ventricular pressure. In addition, gastric ulcer was also considered as a source of upper gastrointestinal bleeding, and therefore, proton pump inhibitors were administered.

After NO therapy (20 ppm) initiation, partial pressure of oxygen/fraction of inspired oxygen increased from 57 to 195 torr. The patient’s respiratory condition gradually improved within 24 h, and she was weaned off mechanical ventilation. She was extubated on Day 6 after admission. Supplemental oxygen was stopped on Day 8, and she was discharged on Day 16. Upper gastrointestinal endoscopy performed 2 weeks after discharge showed a longitudinal linear scar at the gastroesophageal junction (Fig. 1B), which was consistent with the healing process of MWS. Her gastric mucosal findings were normal, and it seemed unlikely that gastric ulcer caused the bleeding. Neither reflux esophagitis nor the separation between the squamocolumnar junction and the diaphragmatic impression were pointed out by upper endoscopy findings. After stopping the administration of proton pump inhibitors, vomiting and hematemesis did not recur. Gastroesophageal reflux and hiatal hernia could be ruled out based on the endoscopic findings and her clinical course. Pathological findings of the gastric and esophageal mucosa showed nonspecific inflammation with mild eosinophilic infiltration, which did not meet the diagnostic criteria for eosinophilic esophagitis.

## DISCUSSION

Hematemesis caused by MWS is a relatively rare condition in early infancy. Back-Lomaniszyn *et al.* reported that MWS accounts for 12.8% of pediatric patients with upper gastrointestinal bleeding [1]. One reason for the rarity of this condition is that children rarely consume alcohol, which is known to trigger MWS [2]. In addition, pathophysiological factors, such as greater tensile circumferential strength of the gastrointestinal tract of infants, may increase their resistance to mucosal laceration [3]. An accurate pathogenesis of MWS in infancy has yet to be elucidated. In our case, there were no risk factors of MWS development, such as alcohol consumption, hiatal hernia or gastroesophageal reflux. However, elevated abdominal pressure caused by heavy lifting or childbirth is a risk factor for MWS [2]. Furthermore, crying or hiccups can elevate abdominal pressure and may cause mucosal tears in infants [4].

Our patient developed severe aspiration pneumonitis due to massive bleeding, which was managed with mechanical ventilation and NO therapy. Patients with aspiration pneumonitis usually recover their respiratory function within 24 h without antibiotics. One study showed that 17% of adult cases with aspiration pneumonitis were treated in intensive care units and 4% died [5]. Few fatal pediatric cases of aspiration pneumonitis have been reported [6]. In one study, aspiration of stomach contents was found in 37% of cases of sudden infant death syndrome [7]. Aspiration in infancy can be a cause of mortality and morbidity. The severity of our case can be attributed to the patient’s young age and inability to turn over, which caused much of the vomited blood to be aspirated into the airway. Although MWS is a rare cause of neonatal hematemesis, patients can become severely ill and require multidisciplinary treatment. Subsequent aspiration pneumonitis can cause severe respiratory failure that requires ventilator management and NO therapy. Notably, an animal study found that high-dose NO therapy may worsen lung damage when used for aspiration pneumonia [8]. However, in this case, NO therapy was necessary because hypoxia and hypercapnia progressed with conventional ventilation. Although no specific side effects were observed after providing NO therapy, careful follow-up examination is necessary to determine the long-term effects. Clinicians should consider MWS as a differential diagnosis when examining an infant who presents with hematemesis and dyspnea.
